# Clinicians’ perceptions of barriers to cervical cancer screening for women living with behavioral health conditions: a focus group study

**DOI:** 10.1186/s12885-022-09350-5

**Published:** 2022-03-09

**Authors:** Rahma S. Mkuu, Stephanie A. Staras, Sarah M. Szurek, Dalila D’Ingeo, Mary A. Gerend, Dianne L. Goede, Elizabeth A. Shenkman

**Affiliations:** 1 Department of Health Outcomes & Biomedical Informatics, University of Florida, 2004 Mowry Rd, Gainesville, FL 32610 USA; 2 College of Medicine, Florida State University, 1115 West Call Street, Tallahassee, FL 32306-4300 USA; 3 Internal Medicine, College of Medicine, University of Florida, 1549 Gale Lemerand Drive, 4th Floor, Suite 4592, Gainesville, FL 32610-3008 USA

**Keywords:** Cervical Cancer Screening, Screening, Cancer prevention, Behavioral health conditions

## Abstract

**Background:**

Women with behavioral health (BH) conditions (e.g., mental illness and substance abuse) receive fewer cervical cancer (CC) screenings, are diagnosed at more advanced cancer stages, and are less likely to receive specialized treatments. The aim of this study was to identify barriers that healthcare providers face in providing CC screening to women with BH conditions.

**Methods:**

Guided by the Consolidated Framework for Implementation Research, we conducted four focus groups in North Florida with 26 primary care and BH clinicians and staff to examine perceived barriers to CC screening among their patients with BH conditions to guide the future development of a tailored cervical cancer screening and follow-up intervention. Thematic analysis was used to analyze verbatim transcripts from audiotaped focus groups.

**Results:**

Three main themes of barriers emerged from the data: 1) BH conditions related barriers included a history of trauma, stigma and discrimination, and uncontrolled comorbid conditions, 2) System level barriers related to lack of integration between BH and primary care, and 3) Similar barriers to the general population including lack of health insurance, insufficient processes to send out reminders, and challenges with communicating with patients.

**Conclusions:**

Tailored CC screening interventions that address the unique needs of women with BH conditions are needed**.** Strategies that address improving trust between patients and healthcare providers, identifying avenues to improve receipt of screening during time-limited clinical visits, connecting BH and primary care providers, and addressing the social determinants of health have potential to improve CC screening rates for women with BH conditions.

## Background

Cervical cancer mortality is preventable through early detection via recommended screening [1]. A significant decline in cervical cancer deaths is largely credited to early diagnosis resulting in identifying cervical abnormalities at the pre-cancer or early cancer stages where treatment is most successful [2, 3]. Despite success in lowering the cervical cancer mortality rate, disparities in cervical cancer mortality continue to persist among underserved populations [4, 5]. Growing research also demonstrates that women living with chronic conditions are less likely to be up to date with cervical cancer screening [6–8].

Mental health and substance use disorders, collectively referred to as behavioral health (BH) disorders by the Substance Abuse and Mental Health Services Administration (SAMHSA) are unfortunately common [9]. People living with behavioral health (BH) conditions (mental health and substance use disorders) are more likely to die of cancer despite having comparable cancer incidence rate to the general population [10, 11]. The lower cancer survival rates among people with BH conditions are likely a result of lower cancer screening rates, leading to more advanced-stage diagnoses [12, 13]. Women with BH conditions are significantly more likely to be diagnosed at later stages and have lower cancer survival rates compared to women without BH conditions [14]. Despite the disparities, a recent systematic review to identify interventions to encourage cancer screening among people with BH conditions found no studies focused on this population [15]. It is imperative to better understand barriers to cervical cancer screening to inform the development of targeted and tailored interventions for women with BH conditions.

Access to care is a well-established barrier for cervical cancer screening, however, despite high rates of healthcare utilization, women with BH conditions do not regularly receive recommended cervical cancer screening [16, 17]. While inconsistent across studies, likely due to the diversity of conditions included within BH conditions, barriers to providing cervical cancer screening to women with BH conditions include low knowledge of cancer among patients, provider negative attitudes towards mental illness, patient challenges with processing information, potential for screening process to exacerbate BH symptoms, poor relationships between staff and patients, travel difficulties, lack of clinician training on how to manage people with BH conditions, and high healthcare costs [18–22]. Moreover, a lack of integration between BH services and primary care leads to uncoordinated care and gaps in knowledge of overdue preventative services which is a systemic barrier to coordinating cancer screenings for individuals with BH conditions [18, 19].

Miller et al., explored challenges to providing both breast and cervical cancer in a community-based healthcare system and two teaching hospitals that provide mental health services. The study’s findings were focused more on breast cancer (mammography) screening barriers than cervical cancer screening barriers [19]. The study highlighted that lack of engagement and communication between BH health and primary care providers was a barrier to providing breast and cervical cancer screening [19]. The study however was conducted in 2007 and did not focus on the mechanisms which serve as a barrier to providing in-clinic cervical cancer screening to women with BH conditions [19].

The aim of this study is to examine provider perspectives on barriers to providing cervical cancer screening for women with BH conditions. The study was informed by the Consolidated Framework for Implementation Research (CFIR), a framework used in implementation science to systematically assess potential barriers and facilitators in the process of developing theoretically informed evidence-based interventions [23, 24]. The CFIR framework has five major domains: intervention characteristics, outer setting, inner setting, characteristics of individuals who are involved, and the process of implementation. Each of the domains contains conceptual elements also known as constructs. For example, the domain of outer setting includes the constructs of patients’ needs and resources, and the domain of inner setting includes constructs of structural characteristics, networks and communications, climate, and culture [24]. The CFIR was used in this study because it provides a pragmatic structure to guide formative research that can be applied when developing interventions.

## Methods

### Study design

This study utilizes qualitative focus group data from a larger mixed method study, Project CONTINUITY: CONnecTing hIgh risk aNd Underserved Individuals To care in the communitY, which aims to identify cancer screening priorities, and develop corresponding cancer screening and linkage to care interventions for women with BH conditions in North Florida. Focus groups with healthcare providers and staff were conducted between June, 2020 and November, 2020 remotely using Zoom because of the Coronavirus Disease (COVID-19) pandemic. Focus groups were conducted at primary care and BH facilities that serve women with BH conditions. We concentrated on primary care providers because they provide an estimated 50% of care for common psychiatric disorders. In addition, we wanted to focus on BH safety net providers because this is a potential avenue to identify women eligible for evidence-based cancer-screenings and to make referrals to primary care providers [25]. The use of focus groups allowed for exploration of personal, relational and collective experiences through group interaction [26].

### Participants

Four focus (FG) groups were conducted among 26 clinicians (e.g., physicians, physician assistants, and nurses) and clinical staff. Purposive convenience sampling was used to recruit healthcare providers who serve women with BH conditions in primary care (academic and community clinics) and behavioral health clinics. One FG of primary care (PC) providers was made up of representatives from three clinics that were part of the same department in a large academic health center. Individuals were recruited after engagement with and permission from each clinic’s leadership. Each focus group ranged in size, (BH FG 1, *n* = 6, BH FG 2, *n* = 5, BH FG 3, *n* = 5, and PC FG 01, *n* = 10). The number of participants was appropriate and manageable in terms of insuring all participants had opportunity to contribute to the discussions.

### Procedure

The University of Florida Health Science Center Institutional Review Board (IRB#202,000,767) approved this study. Focus groups took place during breakfast or lunch hours to minimize interruptions to the clinical workflow. The focus group interview questions were guided by the CFIR, because of its emphasis on examining the influence of personal, organizational, and external factors in the implementation of new interventions [24]. Participants were asked to share barriers to cervical cancer screening and linkage to care specific to serving women with BH conditions. Table 1 outlines the question about barriers along with follow-up probes that were used during the focus group interviews co-author SZ facilitated focus groups with the help of research coordinators.

**Table 1:** Question on barriers to cervical cancer screening and follow-up probes

What are some of the barriers for cervical cancer screening for this vulnerable population?	
Healthcare system (external), clinical (internal) barriers	Insurance/Cost—To what extent is the cost a barrier? Because the insurer might not cover the care?
Infrastructure/Ability:	Reminders/EHR and IT/ time/ priority of cancer vs. other acute problems
Access to services	Women’s access to services including scheduling and transportation? Geographic movement of patients across healthcare systems?
Other Social Determinants of Health for this population?	Cell phone/internet access?
	To what extent is patient trust a barrier for cervical cancer screening?
	Patient knowledge of / prioritizing preventive screenings

### Data analysis

All focus groups were transcribed verbatim and de-identified during transcription. Two authors (RM and SZ) independently coded the transcripts, focusing on participant discussions of barriers to providing cervical cancer screening to women with BH conditions. The study team followed the process of thematic analysis outlined by Clarke and Braun, 2014 [27]. First, the study team familiarized themselves with the data through reading and rereading the transcripts. Then, the domains and constructs of the CFIR were used as a guide to systematically code the data [24]. Coding was conducted in three cycles. First, line by line coding was used to identify quotes that highlighted barriers to screening in each focus group transcript. Second, topic codes were generated by each coder independently. Then, the salient codes characterized by mention in more than 3 focus groups were used to group each of the barriers. The coding team met to resolve discrepancies in coding and interpretations of the data and to discuss salient topic codes. The salient codes were associated with the CFIR domains of outer setting, inner settings, and characteristics of individuals [24]. Although coding was informed by the CFIR domains, we did not limit identified codes and responses from participants to the domains and constructs. After coding was complete, similarities in the coded quotes were identified to find patterns of semantic meaning and concepts. Codes that were related were clustered together to form themes to tell the story.

## Results

### Participants

The majority of participants were female (*n* = 23, 85%), White (*n* = 14, 53.8%), and non-Hispanic (*n* = 23, 88.5%). A large percentage of participants were physicians (*n* = 9, 34.6%), followed by leadership/administrators (*n* = 5, 19.2%), and nurses (*n* = 4, 15.4%). Most had a medical degree (*n* = 10, 38.5%), followed by master’s degree (*n* = 7, 26.9%). Table 2 outlines participant demographic characteristics.

**Table 2:** Demographic Characteristics

	**All *n* = 26**	**Leadership *n* = 5**	**Physicians *n* = 9**	**Nurses (*n*** **= 4)** (APRN = 2, RN = 2)	**Other Clinic Staff**^**a**^ **(*n* = 8)**
**Sex (% female)**	88.5%	100.0%	66.7%	100.0%	100.0%
**Race**					
Asian	11.5%	20.0%	22.2%	–	–
Black or African American	26.9%	20.0%	–	50.0%	5/8
White	57.7%	60.0%	66.7%	50.0%	3/8
Other	3.8%	–	11.1%	–	–
**Ethnicity**					
Hispanic/Latino	3.8%	–	11.1%	–	–
Not Hispanic/Latino	92.3%	100.0%	77.8%	100.0%	100.0%
No response/missing	3.8%	–	11.1%	–	–
**Education**					
Some college	3.8%	–	–	25.0%	–
Associates	3.8%	–	–	25.0%	–
Bachelors	19.2%	–	–	–	5/8
Masters	26.9%	80.0%	–	25.0%	2/8
Doctorate	7.7%	–	–	25.0%	1/8
MD	38.5%	20.0%	100.0%	–	–

^a^Other Clinical Staff: Counselor/health specialists *n* = 3, Case Managers *n* = 3, Social Worker *n* = 1, Clinic Staff *n* = 1

### Themes

The findings from the focus groups are presented for each theme and sub-theme. The quotes from participants are presented verbatim, however, names are redacted to maintain participant confidentiality. Identification codes are presented for each focus group (FG) by type of providers interviewed (BH = behavioral health, PC = primary care). Three main themes emerged from the data: 1) BH conditions related barriers, 2) System level barriers related to BH care, and 3) Similar barriers to the general population. The results are organized by theme. Table 3 outlines CFIR constructs and domains in relation to the themes presented.

**Table 3:** CFIR Constructions in relation to themes presented

Theme	CFIR domain	Sub codes associated with domain	Example Quotes
**CFIR Construct: Characteristics Of Individuals**			
BH conditions related barriers	Other Personal Attributes	•A history of trauma	•* “Some women have – with behavioral health have maybe had traumatic experiences in the past, whether it’s physical or sexual abuse. And so that is a very difficult topic for them to sort of broach and be comfortable with.”(PC_FG_01)*
		•Uncontrolled comorbid conditions	•* “If they are in a situation where they are experiencing severe depression or severe anxiety, they don't have the ability to literally leave the house to get any services or—that's [cervical cancer screening] not their even primary concern.” (BH_FG_01)*
**CFIR Construct: Inner Setting**			
** BH conditions related barriers**	Culture	•Stigma and perceived discrimination	•* “There's absolutely a stigma that's associated with the clients—our clients, our residents seeking outside medical care, and then being open and honest about their history, the fear that maybe a provider will deny to treat them because of that history and the risks associated with it. It's hard. It's most certainly hard.” (BH_FG_01)*
** System level barriers related to BH care**	Structural Characteristics	•Lack of integration between behavioral health and primary care services	•* “Still have trouble at {our primary care site} communicating between that group (BH providers) given some of the firewalls that exist.”(PC_FG_01)*
** Similar barriers to the general population**		•No process to send out reminders	•* “One thing that’s not automatic is letting them (patients) know when the next one (screening) is due.” (PC_FG_01)*
**CFIR Construct: Outer Setting**			
** Similar barriers to the general population**	Patient Needs and Resources	•Lack of health insurance	•* “Well, one definite barrier we see here a lot is the lack of health insurance.” (BH_FG_03)*
		•Communication Challenges	•* “Either we cannot get them on the phone or their phone’s been disconnected, or stuff like that.” (PC_FG_01)*

1. BH conditions related barriers

Healthcare providers cited that patients’ BH conditions are a barrier to providing cervical cancer screening. BH conditions are perceived to impact cervical cancer screening in three ways: (1) a history of trauma is a barrier to communication and results in lack of trust between patients and healthcare providers, (2) stigma and discrimination towards BH conditions prevents patients from seeking primary care services, and (3) uncontrolled BH conditions limits time for preventative services.

#### A history of trauma (CFIR construct: characteristics of individuals)

Trauma was recognized as a barrier to cervical cancer screening by both primary care and BH providers. Women with BH conditions that have a history of sexual trauma, experience stigma and shame associated with their trauma which serves as a barrier to communication about cervical cancer screening. BH providers emphasized the dimension of trauma as a barrier to care.

##### BH providers shared:

Trauma is a really, really - it just follows along with substance use, whether you have it before or it comes from use, trauma is a big part of it. And a lot of our female clients have a lot of sexual trauma, a lot of sexual trauma. And it's really hard for them, you could tell just talking about - they don't want to even - a lot of them, it's hard to even discuss (cervical cancer screening). (BH_FG_01)

##### Another provider added:

And there's a shame associated with it. Going back to a barrier, sometimes it's that self-shame can be a barrier, just don't want to talk about it. The trauma itself could be the barrier. (BH_FG_01)

#### Stigma and discrimination (CFIR Construct: inner setting)

Stigma surrounding mental health was cited as a barrier to accessing preventive cancer screenings. Healthcare providers sited that their patients have recounted experiences of discrimination and mistreatment from non-BH providers. One provider voiced:

… I've had a couple in the past week who've said things like they tried to go to emergency room for something, and they were treated like a "drug addict" and they hated that because they're in a program, they're trying to get help, their lives are in recovery, and yet, because they're on methadone or Buprenorphine or even Vivitrol, they are considered lower or like sub-standard people. I mean it's very sad. And I've heard that throughout my career with this clientele, but specifically recently it's happened a lot. (BH_FG_01)

Stigma and discrimination experienced by patients with BH conditions leads to lack of trust especially with new healthcare providers. One participant added:

.. I think going along with the trust issues, a lot of our patients have schizophrenia or they can be extremely paranoid, so it takes a long time for them to build up trust (*BH_FG_03)*

##### Another provider added:

I would say so. Just in general yes, there can be a mistrust because of things that I'm sure that has happened in the past or something that they have heard from a family member about their experience, that fear comes into play and then you find that there’s mistrust. But I think the biggest and the most important thing when it comes to trying to get our clients out for screening, or trying to do screening overall is, of course, trust must play a big part. But it's more with the clients themselves and their primary care physician. I think the trusts are there. And if the client trusts their primary care physician to order something that is needed, they may have enough trust to move forward getting those screenings... (BH_FG_031)

#### Uncontrolled BH/ chronic conditions (CFIR construct: characteristics of individuals)

Participants with uncontrolled conditions with symptoms like depression or severe anxiety prioritize their acute symptoms over preventative healthcare services like cervical cancer screening. One provider shared how unstable BH conditions can limit patients from accessing healthcare:

I think an additional barrier for quite a number of patients that I've encountered, especially near the beginning of their episode of care with us, is lack of mental healthcare. If they are in a situation where they are experiencing severe depression or severe anxiety, they don't have the ability to literally leave the house to get any services or - that's not their even primary concern. They're just mentally not able to attend to that. Their first need is mental health stability. (BH_FG_01)

When patients do access healthcare services, the management of uncontrolled medication conditions and acute concerns represent urgent priorities overshadowing the need to address preventative services during time-limited visits. One provider stated:

I agree that they [patients with BH conditions] often come in crisis, and the other issue is that the – depending on the severity of their behavioral health issues, the time spent even at a schedule visit, to address not only that but the impact of the behavioral problem on other comorbidities, like diabetes, hypertension consumes much of the time in a visit. And the pelvic exam – and we can get into this as it’s conventionally thought of, is thought of as time consuming and needs preparation. So you really need a perfect – a perfect situation where the behavioral health issue is stable, controlled. The comorbidities are stable and the patient’s – really the key agenda for that visit is yes, it’s a wellness visit or a gynecologic exam visit to get it done. That’s tricky. (PC_FG_01)

Healthcare providers shared that it is challenging to schedule follow-up care for preventive services. One provider shared:

When a patient is here and they have all of these chronic issues and they want to talk about that, they don't necessarily want to say, “Okay, I’ll schedule that the next visit.” And then the next visit comes around and they’ll push it back. So, they will want to push forward. (PC_FG_01)

2) BH specific System level barriers

Healthcare providers shared that lack of integration between BH and primary care services was a barrier to providing or referring patients to cervical cancer screening services.

#### Lack of integration between behavioral health and primary care services (CFIR Construct: inner setting)

The lack of integration and communication between behavioral and primary care services was cited as a barrier to identifying and following up with women in need of screening services. One focus group participant shared:

And I think part of it is a little bit more of an all hands on deck where there is more communication between mental health and primary care, and we, you know, still have trouble at {our primary care site} communicating between that group (BH providers) given some of the firewalls that exist. And I think, you know, oftentimes they’re not thinking about those issues when they’re seeing them, but then that may be the only contact that the particular patient’s getting. They’re kind of off of our radar... (PC_FG_01)

3) Similar Barriers to the general population

Barriers mentioned during focus groups interviews that are similar to documented barriers in the general population included (1) lack of health insurance, (2) no process to remind patients about cervical cancer screening, and (3) challenges communicating with patients.

#### Lack of health insurance (CFIR Construct: outer setting)

Lack of access to health insurance was cited as a barrier to providing cervical cancer screening. One provider shared:

A huge barrier that we see is the financial ability to afford it…[staff member] is one of our uninsured workers. So, she works with a lot of clients that obviously don't have insurance, but it's generally have no job, no income of any sort. (BH_FG_02)

Even among patients who are insured, there is concern of incurring out-of-pocket costs associated with screening. One provider added:

I also have patients who are worried about cost a lot and often I don’t have that information, whether their insurance will cover it. So sometimes they want to call their insurance, which sometimes doesn’t happen. But that’s been a barrier too (PC_FG_01)

#### Lack of adequate process to send reminders (CFIR Construct: inner setting)

Healthcare providers also addressed difficulties in contacting patients to send screening notifications and reminders:

One thing that’s not automatic is letting them [patients] know when the next one [screening] is due. So you asked about how do patients – do patients recognize like when they’re due for it? That’s an area that is provider dependent for us to write that in. (PC_FG_01)

Even for patients who are engaged in care through primary care visits, there is a lack of robust reminder system making it challenging to schedule follow-up visits.

Another thing I would add is sometimes some patients, it seems that they go – each visit is sort of a crisis and you deal with that and then that takes up the entire visit and you sort of get – you lose the health maintenance aspects. And as we’ve already said, you know, our system doesn’t have robust reminders. We have to look to see if people are up to date with things, and sometimes in most settings that gets missed. (PC_FG_01)

#### Communication challenges (CFIR Construct: outer setting)

Healthcare providers shared that some patients’ lack communication resources preventing them from successfully communicating with patients. One provider shared:

Yeah, a lot of times, they won't have cell phones or they get a different cell phone quite frequently or we lose contact. And we have a lot of individuals that are homeless, and by that, I mean, yeah, they're CouchSurfing with different friends. Our homeless shelter's currently close. So, all of those individuals are in hotels in the city and we might lose track of where they're at, how to reach them, especially if you don't have a cell phone. And then, they just don't follow a lot because they can't be reminded of appointments or they can't be called for the specialized to be told, "Hey, you have an appointment on this day at this time. So, it's just the inability to reach them. (BH_FG_02)

Healthcare providers serving patients in rural areas cited lack of access to reliable internet or cell phone service as a barrier to communicating with patients with limited resources.

A lot of our clients don't have access to it [internet]. I mean there are a lot that do, but many people don't have the ability to have even Wi-Fi so they can use like Zoom or anything like that. They're just struggling financially, and they don't have - even in rural areas, especially (BH_FG_01)

## Discussion

This study provides important insight on barriers to providing cervical cancer screening to women with BH conditions in clinical settings. The findings revealed that trauma, uncontrolled BH conditions, stigma and discrimination, lack of integration between BH and primary care, and lack of robust processes to send out reminders are specific barriers to providing women with BH conditions cervical cancer screening. Similar barriers to the general population included lack of health insurance and communication challenges.

A history of trauma, especially sexual trauma, is a barrier to providing cervical cancer screening. A history of sexual trauma is associated with co-occurrence of BH conditions [28]. Women with a history of sexual trauma such as sexual abuse are less likely to be screened for cervical cancer [29]. The lower likelihood of screening is associated with anxiety, fear, and shame associated with the gynecological procedure [30]. Healthcare providers shared that building rapport with patients who have a history of sexual trauma was key to building trust. Trauma informed care and training is needed to improve trust between primary care providers and women with BH conditions [31–33].

Stigma related to BH conditions may influence provider social judgements about patients, negative attitudes and treatment of patients that lead to low prioritization of equitable care [34, 35]. For example, stigma may lead healthcare providers to assume that people with BH conditions may not be able adhere to recommendations (e.g., follow-up if an abnormality is detected) due to their BH conditions [36–38]. People with BH conditions report being the target of stigmatizing attitudes and behaviors related to having a BH diagnosis [39–41]. A study using vignettes of patients found primary care providers had significantly higher negative attitudes (stereotyping and attribution of health challenges to mental illness) towards patients with schizophrenia [42]. The reports of discrimination from healthcare providers explains findings that patients lack trust in their providers [43, 44]. Our findings call for efforts to minimize stigma of BH conditions amongst healthcare providers to help improve trust between patients and healthcare providers [45, 46].

Despite access to general preventative care, utilization of primary care services is not associated with meeting cervical cancer screening recommendations among people with comorbidities [17, 47, 48]. For example, women with comorbidities are diagnosed at later stages of cancer [9, 49–51]. Studies have found rates as high as 80.7% of physical health comorbidities among people with BH conditions [52, 53]. Our study provides an explanation to findings from previous studies. Pre-existing conditions (both BH and chronic medical conditions) are a barrier to screening because addressing uncontrolled symptoms overshadows the ability to perform cervical cancer screening during time-limited visits. The short length of time of primary care visits is associated with decreased prioritization and provision of screening for several conditions including blood pressure, depression, and cancer [54–56]. There is a need to address the time constraint barrier that prevents healthcare providers from conducting preventative services during time-limited clinical visits.

Healthcare providers shared that a lack of integration between BH and primary care services, a lack of a system to identify women who are due for screening, lack of follow-up, and lack of resources to support women with limited access to care were barriers to providing cervical cancer screening. Integration between BH care and primary care is associated with improving concordance of chronic disease treatment and receipt of preventative health services including cancer screening among people with BH conditions [57]. Since cervical cancer screening is a service provided at primary care settings, integration allows communication between BH providers and primary care to facilitate identifying women in need of screening or due for follow-up care.

Out of pocket expenses and the lack of access to health insurance are well cited structural barriers to cancer screening [58, 59]. For patients with BH conditions, lack of health insurance is a major barrier to care [60]. Medicaid is a major insurer for people with BH conditions, however the state of Florida did not expand Medicaid eligibility [61]. In addition to lack of access to health insurance, limited charity care resources in the area further exacerbate lack of access to screening services. Even for patients with insurance, healthcare providers shared that patients were not aware of their plan coverage and worried that their insurance would not cover the screening. Patients were also reported to experience challenges with contacting their insurance about coverage questions highlighting a need to assist patients with navigating and understanding their insurance benefits. The Florida Breast and Cervical Cancer Early Detection Program (FBCCEDP) should be recommended to uninsured eligible women as a free or low cost avenue to access screening [62]. Women may have to travel long distances to access the FBCCEDP as the program is only available in specific counties [62]. Supporting evidence supports our findings that clinicians lack systems to support identification of patients who are due for cervical screening and follow-up [63]. Patient reminders in the form of letters, text messages, using mobile applications are strategies that have been found to increase cervical cancer screening and follow-up rates [64–66]. Provision of communication tools alone is insufficient to overcoming barriers to communicating. Patient communication preferences, level of digital literacy, and stable housing need to be considered in interventions that address communication challenges between patients and healthcare providers [67–69].

Although our study contributes to the scarce research soliciting provider identified barriers to cervical cancer screening, findings must be considered with respect to some limitations. First, we conducted a total of four focus groups a higher number of focus groups would likely provide more contextual understanding of barriers [70]. The use of purposive sampling was both a strength and weakness of this study. Purposive sampling was a weakness because it limited our ability to examine variability across a wide range of types of clinical practices (e.g., emergency room departments, urgent care facilities). Purposive sampling was a strength because our participants included nurses, caseworkers, and others who regularly provide care to patients with BH conditions resulting in facilitating a broader understanding from a diverse group of healthcare providers who serve patients in settings with similar clinical workflow allowing for the development of targeted interventions for the specific clinical practices in the future. Difficulties with recruiting diverse provides coupled with the voluntary nature of participation of healthcare providers may have resulted in self-selection bias therefore results are limited to providers from the clinics that participated in the focus groups. The group dynamics during focus group may have influenced participant responses [71]. We limited “group think” by soliciting additional contributions from each participant and encouraged participants to share novel responses as well as build upon existing conversations. Lastly, while lack of insurance coverage was identified as a barrier to cervical cancer screening by study participants, we did not review the patient insurance demographics of the participating providers. Qualitative studies are important for understanding context, increasing understanding, and generating hypotheses. Future work should improve upon the sampling and methodological approach and include perspectives from both healthcare providers and patients.

## Implications for practice

Designed interventions to improve cervical cancer screening among women with BH conditions should consider strategies to improve trust between patients and providers, support BH and chronic condition management, and improve communication systems that facilitate continuity of care. The use of the CFIR allows for articulation of findings using a standardized common language and list of constructs and domains that can serve as a guide in the development of a future theory-driven targeted cervical cancer screening intervention for women with BH conditions. The results of this study can also be applied as building blocks for developing future hypothesis driven studies informed by the standardized CFIR theoretical framework [72]. For example, CFIR has been used to identify, adapt, implement, and evaluate evidence-based actionable strategies to improve colorectal cancer [73–75].

## Conclusions

Findings highlight the need for tailored interventions that address both clinic and patient barriers to cervical cancer screening for women with BH conditions. Multilevel interventions that address social determinants of health and are sensitive to the patients’ BH needs are needed to facilitate both cervical cancer screening and follow-up with recommended care.

## Data Availability

The datasets supporting conclusions of this article are not available publically bit are available from the study PI: Elizabeth Shenkman on reasonable request.

## Abbreviations

BH: Behavioral Health
CC: Cervical Cancer
CFIR: Consolidated Framework for Implementation Research
FBCCEDP: Florida Breast and Cervical Cancer Early Detection Program
FG: Focus Group
PC: Primary Care

## References

[1] Centers for Disease Control and Prevention (CDC). 2021. What Can I Do to Reduce My Risk of Cervical Cancer? Accessed June 1, 2021. https://www.cdc.gov/cancer/cervical/basic_info/prevention.htm

[2] Centers for Disease Control and Prevention (CDC). 2020. Cervical Cancer Statistics. Accessed June 1, 2021. https://www.cdc.gov/cancer/cervical/statistics/index.htm

[3] Fontham ETH, Wolf AMD, Church TR, Etzioni R, Flowers CR, Herzig A, Guerra CE, Oeffinger KC, Shih Y-CT, Walter LC, Kim JJ, Andrews KS, DeSantis CE, Fedewa SA, Manassaram-Baptiste D, Saslow D, Wender RC, Smith RA (2020). Cervical cancer screening for individuals at average risk: 2020 guideline update from the American Cancer Society. CA Cancer J Clin.

[4] Gauri A, Messiah SE, Bouzoubaa LA, Moore KJ, Koru-Sengul T (2020). Cervical cancer sociodemographic and diagnostic disparities in Florida: A population-based study (1981–2013) by stage at presentation. Ethn Health.

[5] Simard EP, Fedewa S, Ma J, Siegel R, Jemal A (2012). Widening socioeconomic disparities in cervical cancer mortality among women in 26 states, 1993–2007. Cancer.

[6] Liu BY, O’Malley J, Mori M, Fagnan LJ, Lieberman D, Morris CD, Buckley DI, Heitzman JD, Carney PA (2014). The Association of Type and Number of Chronic Diseases with Breast, Cervical and Colorectal Cancer Screening in Rural Primary Care Practices. J Am Board Fam Med.

[7] Bhatia D, Lega IC, Wu W, Lipscombe LL (2020). Breast, cervical and colorectal cancer screening in adults with diabetes: A systematic review and meta-analysis. Diabetologia.

[8] Murphy KA, Stone EM, Presskreischer R, McGinty EE, Daumit GL, Pollack CE (2021). Cancer Screening Among Adults With and Without Serious Mental Illness: A Mixed Methods Study. Med Care.

[9] Center for Behavioral Health Statistics and Quality. Behavioral health equity report 2021: Substance use and mental health indicators measured from the National Survey on Drug Use and Health (NSDUH), 2015–2019. 2021; Publication No. PEP21–07–01–004. Rockville, MD: Substance Abuse and Mental Health Services Administration. Accessed December 22, 2021. https://www.samhsa.gov/data/

[10] Kisely S, Crowe E, Lawrence D (2013). Cancer-related mortality in people with mental illness. JAMA Psychiat.

[11] Zhuo C, Tao R, Jiang R, Lin X, Shao M (2017). Cancer mortality in patients with schizophrenia: Systematic review and meta-analysis. Br J Psychiatry.

[12] Woodhead C, Cunningham R, Ashworth M, Barley E, Stewart RJ, Henderson MJ (2016). Cervical and breast cancer screening uptake among women with serious mental illness: A data linkage study. BMC Cancer.

[13] Solmi M, Firth J, Miola A, Fornaro M, Frison E, Fusar-Poli P, Dragioti E, Shin JI, Carvalho AF, Stubbs B, Koyanagi A, Kisely S, Correll CU (2020). Disparities in cancer screening in people with mental illness across the world versus the general population: Prevalence and comparative meta-analysis including 4 717 839 people. Lancet Psychiatry.

[14] Davis LE, Bogner E, Coburn NG, Hanna TP, Kurdyak P, Groome PA, Mahar AL (2020). Stage at diagnosis and survival in patients with cancer and a pre-existing mental illness: A meta-analysis. J Epidemiol Community Health.

[15] Barley EA, Borschmann RD, Walters P, Tylee A (2013). Interventions to encourage uptake of cancer screening for people with severe mental illness. Cochrane Database Syst Rev.

[16] James M, Thomas M, Frolov L, Riano NS, Vittinghoff E, Schillinger D, Newcomer JW, Mangurian C (2017). Rates of Cervical Cancer Screening Among Women with Severe Mental Illness in the Public Health System. Psychiatr Serv.

[17] Abrams MT, Myers CS, Feldman SM, Boddie-Willis C, Park J, McMahon RP, Kelly DL (2012). Cervical cancer screening and acute care visits among Medicaid enrollees with mental and substance use disorders. Psychiatr Serv.

[18] Clifton A, Burgess C, Clement S, Ohlsen R, Ramluggun P, Sturt J, Walters P, Barley EA (2016). Influences on uptake of cancer screening in mental health service users: A qualitative study. BMC Health Serv Res.

[19] Miller E, Lasser KE, Becker AE (2007). Breast and cervical cancer screening for women with mental illness: Patient and provider perspectives on improving linkages between primary care and mental health. Arch Womens Ment Health.

[20] Aggarwal A, Pandurangi A, Smith W (2013). Disparities in breast and cervical cancer screening in women with mental illness: a systematic literature review. Am J of Preventive Med.

[21] Moravac CC (2018). Reflections of homeless women and women with mental health challenges on breast and cervical cancer screening decisions: power, trust, and communication with care providers. Front Public Health.

[22] Clifton A, Burgess C, Clement S, Ohlsen R, Ramluggun P, Sturt J, Barley EA (2016). Influences on uptake of cancer screening in mental health service users: a qualitative study. BMC health services research.

[23] Breimaier HE, Heckemann B, Halfens RJG, Lohrmann C (2015). The Consolidated Framework for Implementation Research (CFIR): A useful theoretical framework for guiding and evaluating a guideline implementation process in a hospital-based nursing practice. BMC Nurs.

[24] Damschroder LJ, Aron DC, Keith RE, Kirsh SR, Alexander JA, Lowery JC (2009). Fostering implementation of health services research findings into practice: A consolidated framework for advancing implementation science. Implement Sci.

[25] National Institute of Mental Health. Integrated care. 2017. https://www.nimh.nih.gov /health/topics/integrated-care/index.shtml. Accessed 11 Jan 2022.

[26] Morgan DL (1996). Focus Groups. Annu Rev Sociol.

[27] Clarke, V., & Braun, V. Thematic Analysis. In T. Teo (Ed.), Encyclopedia of Critical Psychology (pp. 1947–1952). Springer. 2014. 10.1007/978-1-4614-5583-7_311

[28] McHugo GJ, Caspi Y, Kammerer N, Mazelis R, Jackson E, Russell L, Clark C, Liebschutz J, Kimerling R (2005). The assessment of trauma history in women with Co-occurring substance abuse and mental Disorders and a history of interpersonal Violence. J Behav Health Serv Res.

[29] Farley M, Golding JM, Minkoff JR (2002). Is a history of trauma associated with a reduced likelihood of cervical cancer screening?. Fam. Pract..

[30] Cadman L, Waller J, Ashdown-Barr L, Szarewski A (2012). Barriers to cervical screening in women who have experienced sexual abuse: An exploratory study. J Fam Plann Reprod Health Care.

[31] Purkey E, Patel R, Phillips SP (2018). Trauma-informed care: Better care for everyone. Can Fam Physician.

[32] Reeves E (2015). A Synthesis of the Literature on Trauma-Informed Care. Issues Ment Health Nurs.

[33] Covington SS (2008). Women and Addiction: A Trauma-Informed Approach. J Psychoactive Drugs.

[34] Perry A, Lawrence V, Henderson C (2020). Stigmatisation of those with mental health conditions in the acute general hospital setting. A qualitative framework synthesis. Soc Sci Med.

[35] Ewart SB, Bocking J, Happell B, Platania-Phung C, Stanton R (2016). Mental Health Consumer Experiences and Strategies When Seeking Physical Health Care: A Focus Group Study. Glob Qual Nurs Res.

[36] Thornicroft G, Rose D, Kassam A (2007). Discrimination in health care against people with mental illness. Int Rev Psychiatry.

[37] Vistorte AOR, Ribeiro WS, Jaen D, Jorge MR, Evans-Lacko S, de J Mari J (2018). Stigmatizing attitudes of primary care professionals towards people with mental disorders: A systematic review. Int J Psychiatry Med.

[38] van Boekel LC, Brouwers EPM, van Weeghel J, Garretsen HFL (2013). Stigma among health professionals towards patients with substance use disorders and its consequences for healthcare delivery: Systematic review. Drug Alcohol Depend.

[39] Ross LE, Vigod S, Wishart J, Waese M, Spence JD, Oliver J, Chambers J, Anderson S, Shields R (2015). Barriers and facilitators to primary care for people with mental health and/or substance use issues: A qualitative study. BMC Fam Pract.

[40] Spear SE, Shedlin M, Gilberti B, Fiellin M, McNeely J (2016). Feasibility and acceptability of an audio computer-assisted self-interview version of the Alcohol, Smoking and Substance Involvement Screening Test (ASSIST) in primary care patients. Substance Abuse.

[41] McNeely J, Kumar PC, Rieckmann T, Sedlander E, Farkas S, Chollak C, Kannry JL, Vega A, Waite EA, Peccoralo LA, Rosenthal RN, McCarty D, Rotrosen J (2018). Barriers and facilitators affecting the implementation of substance use screening in primary care clinics: A qualitative study of patients, providers, and staff. Addict Sci Clin Pract.

[42] Mittal D, Corrigan P, Sherman MD, Chekuri L, Han X, Reaves C, Mukherjee S, Morris S, Sullivan G (2014). Healthcare providers’ attitudes toward persons with schizophrenia. Psychiatr Rehabil J.

[43] Saunders EC, Moore SK, Walsh O, Metcalf SA, Budney AJ, Cavazos-Rehg P, Scherer E, Marsch LA (2021). “It’s way more than just writing a prescription”: A qualitative study of preferences for integrated versus non-integrated treatment models among individuals with opioid use disorder. Addict Sci Clin Pract.

[44] Pugh M, Perrin PB, Rybarczyk B, Tan J (2021). Racism, Mental Health, Healthcare Provider Trust, and Medication Adherence Among Black Patients in Safety-Net Primary Care. J Clin Psychol Med Settings.

[45] Gilson L (2003). Trust and the development of health care as a social institution. Soc Sci Med.

[46] Cockroft JD, Adams SM, Bonnet K, Matlock D, McMillan J, Schlundt D (2019). “A scarlet letter”: Stigma and other factors affecting trust in the health care system for women seeking substance abuse treatment in a community setting. Subst Abus.

[47] Salsberry PJ, Chipps E, Kennedy C (2005). Use of general medical services among Medicaid patients with severe and persistent mental illness. Psychiatr Serv.

[48] Weitlauf J, Jones S, Xu X, Finney JW, Moos RH, Sawaya GF, Frayne SM (2013). Receipt of Cervical Cancer Screening in Female Veterans: Impact of Posttraumatic Stress Disorder and Depression. Women’s Health Issues.

[49] Cunningham R, Sarfati D, Stanley J, Peterson D, Collings S (2015). Cancer survival in the context of mental illness: A national cohort study. Gen Hosp Psychiatry.

[50] Irwin KE, Henderson DC, Knight HP, Pirl WF (2014). Cancer care for individuals with schizophrenia. Cancer.

[51] Soccio J, Brown M, Comino E, Friesen E (2015). Pap smear screening, pap smear abnormalities and psychosocial risk factors among women in a residential alcohol and drug rehabilitation facility. J Adv Nurs.

[52] Osborne B, Kelly PJ, Larance B, Robinson LD, Ivers R, Deane FP, Webber A, Kelly D (2020). Substance Use and Co-occurring Physical Health Problems: File Review of a Residential Drug and Alcohol Treatment Service. J Dual Diagn.

[53] Keaney F, Gossop M, Dimech A, Guerrini I, Butterworth M, Al-Hassani H, Morinan A (2010). Physical health problems among patients seeking treatment for substance use disorders: A comparison of drug dependent and alcohol dependent patients. J Subst Use.

[54] Eaton J, Reed D, Angstman KB, Thomas K, North F, Stroebel R, Tulledge-Scheitel SM, Chaudhry R (2012). Effect of visit length and a clinical decision support tool on abdominal aortic aneurysm screening rates in a primary care practice. Int J Clin Pract Suppl.

[55] Zhang JJ, Rothberg MB, Misra-Hebert AD, Gupta NM, Taksler GB (2020). Assessment of Physician Priorities in Delivery of Preventive Care. JAMA Netw Open.

[56] Schmitt MR, Miller MJ, Harrison DL, Touchet BK (2010). Relationship of depression screening and physician office visit duration in a national sample. Psychiatr Serv.

[57] Murphy KA, Daumit GL, Bandara SN, Stone EM, Kennedy-Hendricks A, Stuart EA, Pollack CE, McGinty EE (2020). Association Between the Maryland Medicaid Behavioral Health Home Program and Cancer Screening in People With Serious Mental Illness. Psychiatr Serv.

[58] Harper DM, Plegue M, Harmes KM, Jimbo M, SheinfeldGorin S (2020). Three large scale surveys highlight the complexity of cervical cancer under-screening among women 45–65years of age in the United States. Prev Med.

[59] Sabatino SA, Thompson TD, White MC, Shapiro JA, de Moor J, Doria-Rose VP, Clarke T, Richardson LC (2021). Cancer Screening Test Receipt—United States 2018. MMWR.

[60] Han B, Compton WM, Blanco C, Colpe LJ (2017). Prevalence, Treatment, And Unmet Treatment Needs Of US Adults With Mental Health And Substance Use Disorders. Health Aff.

[61] Sommers BD, Epstein AMUS (2013). Governors and the Medicaid Expansion—No Quick Resolution in Sight. NEJM.

[62] Florida Department of Health. 2021. Breast and Cervical Cancer Early Detection Program. Accessed December 14, 2021. http://www.floridahealth.gov/diseases-and-conditions/cancer/breast-cancer/bccedp.html

[63] Schapira MM, Sprague BL, Klabunde CN, Tosteson ANA, Bitton A, Chen JS, Beaber EF, Onega T, MacLean CD, Harris K, Howe K, Pearson L, Feldman S, Brawarsky P, Haas JS, PROSPR consortium (2016). Inadequate Systems to Support Breast and Cervical Cancer Screening in Primary Care Practice. J Gen Intern Med.

[64] Bhochhibhoya S, Dobbs PD, Maness SB (2021). Interventions using mHealth strategies to improve screening rates of cervical cancer: A scoping review. Prev Med.

[65] Albrow R, Blomberg K, Kitchener H, Brabin L, Patnick J, Tishelman C, Törnberg S, Sparén P, Widmark C (2014). Interventions to improve cervical cancer screening uptake amongst young women: A systematic review. Acta Oncol.

[66] MacLaughlin KL, Kessler ME, KomandurElayavilli R, Hickey BC, Scheitel MR, Wagholikar KB, Liu H, Kremers WK, Chaudhry R (2018). Impact of Patient Reminders on Papanicolaou Test Completion for High-Risk Patients Identified by a Clinical Decision Support System. J Women's Health.

[67] Anthony DL, Campos-Castillo C, Lim PS (2018). Who Isn’t Using Patient Portals and Why? Evidence and Implications from a National Sample of US Adults. Health Aff.

[68] Asgary R, Naderi R, Wisnivesky J (2017). Opt-Out Patient Navigation to Improve Breast and Cervical Cancer Screening Among Homeless Women. J Womens Health.

[69] Walker DM, Hefner JL, Fareed N, Huerta TR, McAlearney AS (2019). Exploring the Digital Divide: Age and Race Disparities in Use of an Inpatient Portal. Telemed J E Health.

[70] Carlsen B, Glenton C (2011). What about N? A methodological study of sample-size reporting in focus group studies. BMC Med Res Methodol.

[71] Seal DW, Bogart LM, Ehrhardt AA (1998). Small group dynamics: The utility of focus group discussions as a research method. Group Dyn Theory Res Pract.

[72] Kirk MA, Kelley C, Yankey N, Birken SA, Abadie B, Damschroder L (2016). A systematic review of the use of the Consolidated Framework for Implementation Research. Implement Sci.

[73] Cole AM, Esplin A, Baldwin LM (2015). Adaptation of an evidence-based colorectal cancer screening program using the consolidated framework for implementation research. Prev Chronic Dis..

[74] Lam H, Quinn M, Cipriano-Steffens T, Jayaprakash M, Koebnick E, Randal F, Kim K (2021). Identifying actionable strategies: using Consolidated Framework for Implementation Research (CFIR)-informed interviews to evaluate the implementation of a multilevel intervention to improve colorectal cancer screening. Implement Sci Commun.

[75] Kegler MC, Beasley DD, Liang S, Cotter M, Phillips E, Hermstad A, Riehman K (2018). Using the consolidated framework for implementation research to understand safety net health system efforts to increase colorectal cancer screening rates. Health Educ Res.

